# Manipulating rumen microbiome and fermentation through interventions during early life: a review

**DOI:** 10.3389/fmicb.2015.01133

**Published:** 2015-10-14

**Authors:** David R. Yáñez-Ruiz, Leticia Abecia, Charles J. Newbold

**Affiliations:** ^1^Estación Experimental del Zaidín – Consejo Superior de Investigaciones CientíficasGranada, Spain; ^2^Institute of Biological, Environmental and Rural Sciences, Aberystwyth UniversityAberystwyth, UK

**Keywords:** early life, microbial colonization, rumen development, rumen microbiome, weaning

## Abstract

The nutritional manipulations of the rumen microbiome to enhance productivity and health are rather limited by the resilience of the ecosystem once established in the mature rumen. Based on recent studies, it has been suggested that the microbial colonization that occurs soon after birth opens a possibility of manipulation with potential to produce lasting effects into adult life. This paper presents the state-of-the-art in relation to early life nutritional interventions by addressing three areas: the development of the rumen as an organ in regards to the nutrition of the new-born, the main factors that determine the microbial population that first colonizes and establishes in the rumen, and the key immunity players that contribute to shaping the commensal microbiota in the early stage of life to understand host-microbiome specificity. The development of the rumen epithelium and muscularization are differently affected by the nature of the diet and special care should be taken with regards to transition from liquid (milk) to solid feed. The rumen is quickly colonized by all type of microorganisms straight after birth and the colonization pattern may be influenced by several factors such as presence/absence of adult animals, the first solid diet provided, and the inclusion of compounds that prevent/facilitate the establishment of some microorganisms or the direct inoculation of specific strains. The results presented show how early life events may be related to the microbial community structure and/or the rumen activity in the animals post-weaning. This would create differences in adaptive capacity due to different early life experiences and leads to the idea of microbial programming. However, many elements need to be further studied such as: the most sensitive window of time for interventions, the best means to test long term effectiveness, the role of key microbial groups and host-immune regulations.

## Introduction

The forestomachs of ruminant animals contain a great diversity of prokaryotic (bacteria, archaea, virus) and eukaryotic (protozoa and fungi) micro-organisms that together breakdown and ferment the feed ingested by the host animal (Dehority, 2003). In the last decades there have been significant efforts to develop compounds that may shift the rumen fermentation toward more efficient metabolic pathways by targeting key groups of microorganisms (i.e., archaea in case of methanogenesis, Hart et al., 2008). However, the utility of such compounds often appears limited as results are often inconsistent or short-lived. This is mainly due to the difficulty in modifying a well-established and fully matured microbial ecosystem in the rumen of adult animals. There is ample evidence of a strong host-microbiota specificity (Kittelmann et al., 2014), implying that after any alteration (i.e., rumen digesta swapping, exogenous bacteria application or antibiotic treatment), once ceased, the microbial community composition and fermentation profile will return to the original pre-treatment composition (Weimer et al., 2010). The developing rumen in the new-born provides a unique opportunity for potential manipulation of such a complex microbial ecosystem.

Early experience ingesting feeds increases preference for and later consumption of those feeds by animals (Provenza and Balph, 1990). Early dietary experiences have a greater and more lasting effect than those occurring later in life (Distel et al., 1994). Different processes (neurological, morphological, and physiological) may be involved during early in life and can be altered so that animals can better manage in the environment in which they are reared from birth.

Li et al. (2012), based on 454-pyrosequencing of 16S rDNA, reported that a total of 170 bacterial genera exists in the developing rumen of 14 days old calves, and that the microbiota was responsive to dietary modifications as well as physiological changes in the host. Earlier work reported that forage or concentrate diets fed around weaning had an impact on the bacterial population that established in the rumen (Eadie et al., 1959); however, the impact that this differentiation might have later in life on the rumen microbial ecosystem remained to be determined. Recent studies (Yáñez-Ruiz et al., 2010; Abecia et al., 2013, 2014a) suggested that it would indeed be possible to promote different microbial populations establishing in the rumen of the young animal by manipulating the feeding management early in life that persisted in later life. This would create differences in adaptive capacity due to different early life experiences, leading to the idea of microbial programming. However, despite significant research effort, there is still a lack of understanding of the mechanisms governing microbial/host cell interactions, the development of the rumen and its microbial community, and the implications for the host when microbial colonization patterns are altered, especially the long-term effects. This paper will critically review the information published on: (i) the development of the rumen as an organ in regards to the nutrition of the new-born, (ii) the factors (maternal, dietary, etc.) that determine the microbial population that first colonizes and establishes in the rumen and (iii) the key immunity players that contribute to shaping the commensal microbiota in the early stage of life to understand host-microbiome specificity. The aim of the review is to evaluate the importance of the multiple factors in shaping the rumen microbiome and the potential of early life rumen microbial programming based on current research and to identify gaps of knowledge for future research studies.

## The Development of the Rumen as an Organ and the Influence of the Diet

Young ruminants present at birth an undeveloped reticulo-rumen, therefore, until the system is fully matured they function as monogastrics fed on milk-based diets that are not digested in the rumen but in the abomasum (Church, 1988; Davis and Drackley, 1998). As stated by Heinrichs (2005) ‘a smooth transition from a monogastric to ruminant animal, with minimal loss in growth, requires the development of the reticulo-rumen and its associated microbial population for efficient utilization of dry and forage-based diets’.

Development of the rumen is an important physiological challenge for young ruminants (Jiao et al., 2015). It entails growth and cellular differentiation of the rumen, and results in a major shift in the pattern of nutrients being delivered to the intestines and liver, and thus the peripheral tissues of the animal (Baldwin et al., 2004). The development of the rumen involves three distinct processes: (i) anatomical development (growth in rumen mass and growth of rumen papillae; Reynolds et al., 2004), (ii) functional achievement (fermentation capacity and enzyme activity; Rey et al., 2012; Faubladier et al., 2013), and (iii) microbial colonization (bacteria, fungi, methanogens, and protozoa; Fonty et al., 1987; Fouts et al., 2012). This section addresses the first process, while microbial colonization will be discussed in Section “Factors that Influence the Microbiota Establishing in the Rumen and Long Term Effects.”

An inadequate development of the rumen will affect nutrient digestion and absorption (Baldwin et al., 2004). On the other hand, a complete development of the rumen facilitates digestion of feed components, which provides nutrients for the physiological requirements of the animal. The anatomical development of the rumen is a process that occurs following three phases: non-rumination (0–3 weeks); transitional phase (3–8 weeks), and rumination (from 8 weeks on; Wardrop and Coombe, 1960; Lane et al., 2002).

During the transition from a pre-ruminant to a ruminant animal, growth and development of the ruminal absorptive surface area (papillae) is essential to enable absorption and utilization of digestion end products, specifically rumen volatile fatty acids (Warner et al., 1956). The presence and absorption of volatile fatty acids stimulates rumen epithelial metabolism and may be key in initiating rumen epithelial development (Baldwin and McLeod, 2000). Different studies (Nocek et al., 1984; Greenwood et al., 1997) have shown that ingestion of dry feeds and the resultant microbial end products stimulate the development of the rumen epithelium. However, different volatile fatty acids stimulate such development differently, as butyrate is most stimulatory, followed by propionate. With decreasing rumen pH and increasing butyrate concentrations, butyrate metabolism by the epithelium increases concomitantly (Baldwin and McLeod, 2000). A continuous exposure to volatile fatty acids maintains rumen papillae growth, size, and function (Warner et al., 1956). Thus, it is expected that diets consisting of milk, concentrates, or forages affect the rumen epithelial growth to different extents (**Table 1**). Moreover, the establishment and activity of the rumen epithelial tissue-associated microbes (defined as epimural community) may be another factor that influences the extent of development of the rumen epithelium (Malmuthuge et al., 2012, see “Factors that Influence the Microbiota Establishing in the Rumen and Long Term Effects”).

**Table 1:** Effect of different dietary interventions in early life on rumen development parameters.

Dietary treatment	Rumen weight, kg	Wall thickness, cm	Papillae, n/cm^2^	pH	Study
Corn processing	ND	Affected (1.06–1.21)	ND	Affected (5.41–5.66)	Lesmeister and Heinrichs, 2004
Supplemental yeast	ND	No effect	ND	ND	Lesmeister et al., 2004
Supplemental molasses	ND	No effect	ND	ND	Lesmeister and Heinrichs, 2005
Carbohydrate composition	Increased (0.73–1.73)	Increased (0.86–1.32)		Affected (4.9–5.3)	Suárez et al., 2006
Milk allowance	Increased (1.37–1.89)	Increased (1.15–1.47)	Increased (71–86)	Decreased (6.22–5.66)	Khan et al., 2007
Milk allowance	Affected (0.58–1.35)	ND	ND	Affected (5.56–6.29)	Kristensen et al., 2007
Forage to concentrate ratio	Affected (0.95–1.45)	Affected (0.82–1.20)	ND	Affected (5.09–5.23)	Suárez et al., 2007
Starch sources	Affected (1.21–1.53)	Affected (1.55–1.95)	Affected (70–91)	Affected (5.46–5.79)	Khan et al., 2008
Provision of hay	Increased (1.59–1.89)	No effect	No effect	Increased (5.06–5.49)	Khan et al., 2011
Whole milk vs. milk replacer	No effect	ND	ND	No effect	Górka et al., 2011
Whole milk vs. milk replacer	Decreased (0.73–0.66)	ND	ND	Increased (6.12–6.57)	Abecia et al., 2014b
Milk replacer feeding strategy	No effect	ND	ND	Affected (6.2–6.9)	Silper et al., 2014

*ND, not determined*.

The chemical composition of the liquid (milk) feed and the effect of the oesophageal groove limit the process of physical and functional development of the rumen (Warner et al., 1956). In young ruminants fed only milk or milk replacer, the rumen development has been shown to be limited even up to 12 weeks of age (Tamate et al., 1962). Indeed, it has been reported a regression of rumen development when calves were changed from a solid diet and milk replacer to a solely milk/milk replacer diet (Harrison et al., 1960). Also, young ruminants receiving only milk/milk replacer had limited metabolic activity in the rumen epithelium and minimal absorption of volatile fatty acids (Heinrichs, 2005). Therefore, although milk based diet promote rapid and efficient growth of the young animal, it does not contribute to prepare the pre-ruminant to utilize solid diets.

Unlike liquid feeds, solid feeds are mainly directed to the reticulo-rumen for digestion (Church, 1988). Solid feed intake stimulates rumen microbial proliferation and production of volatile fatty acids, which have been shown to initiate rumen epithelial development, although, different solid feeds may differ in their ability to stimulate the development of the rumen. Both the chemical composition of feeds and the resultant microbial digestion end products have the greatest influence on the development of the rumen epithelium (Nocek et al., 1984).

Providing natural milk or milk replacer to newborn ruminants differs not only in their intrinsic differences in nutrient composition but also in the presence or absence of the dam. In ruminant farming two main systems for managing the young animals can be identified. In commercial dairy systems, calves are typically separated from the dam at a young age and fed either milk replacer or whole milk; on the contrary, in fattening systems, the newborns remain with the mother until weaning. It has been recently reported that kid goats reared with the dam had greater rumen development than their twins that were fed on milk replacer and isolated from adult animals, despite both groups having access to the same forage and concentrate offered *ad libitum* (Abecia et al., 2014b). This is accordance with De Paula Vieira et al. (2012), which showed that calves reared in the presence of older companions exhibited more frequent and longer visits to the feeder, which they hypothesized to be a consequence of social learning (Galef and Giraldeau, 2001). However, the advantage of the direct microbial inoculation through physical contact with the dam deserves further attention, as discussed in the following section.

In intensive farming, the supplementation with concentrates is the most common method of providing nutrients to the animal with emphasis on offering young ruminants concentrate solid starter at a relative early age (Jiao et al., 2015). Therefore, in the last years, research on rumen development has been mainly directed on this type of feeding system and the main factors that affect rumen development in ruminants fed a range of different diets (Owens et al., 1993), with the primary attention on diet composition (**Table 1**, Coverdale et al., 2004; Suárez et al., 2007; Khan et al., 2011). Feeding concentrate feeds in early life stimulates the development of the epithelium, while forages with large particle size or high fiber sources appear to be the primary stimulators of rumen muscularization and volume (Zitnan et al., 1998). Several recent studies have shown that another effective method to foster solid feed intake in calves, contrary to what it has been traditionally adviced, is to provide *ad libitum* access to poor quality (nutritionally) chopped straw or hay (Jiao et al., 2015). Castells et al. (2013) conducted a meta-analysis and concluded that there were no differences in gut fill between calves consuming no forage and calves consuming forage up to 5% of total solid feed consumption. Thus, it can be concluded that when forage consumption is less than 5% of the total solid feed intake, gut fill is negligible and thus advantages reported in performance and efficiency when feeding chopped forages to calves are not an artifact due to gut fill. Depriving calves from forage during the pre-weaning phase may offer yet another physiological and dietary adaptation challenge to young calves during the transition when presented with forage for the first time. Phillips (2004) reported that calves fed fresh grass during the milk-feeding period spent more time eating on a pasture compared with those that received no forage before weaning. Recent data also shows that 22% of the variation in milk yield in first lactation is associated to the average daily gain during the first weeks of life (Soberon et al., 2012). However, the long-term effects of early life nutritional management in relation to rumen development are still largely unknown and there are factors that still need to be carefully considered such as composition of the starter, type of forage and timing of its introduction.

When addressing the development of the rumen, the following question arises: does the development of the organ determine which microbes colonize the rumen or do the microbes themselves shape the rumen development through their activity and specific signaling? In the adult animal, the diet is the main driver of the microbial community structure (McCann et al., 2014), but in the pre-ruminant both microbial colonization and rumen development may interact in a way that one influences the other. Also, it is still unknown to what extent the animal is genetically pre-determined to develop a certain type of rumen (i.e., epithelium, muscularization, contractions). Goopy et al. (2012) reported that low methane yield sheep were associated with a shorter mean retention time of particulate and liquid digesta, lesser amounts of rumen particulate content and a smaller rumen volume. Low methane yield sheep harbor a distinctive bacterial community structure (Kittelmann et al., 2014). Thus, it could be hypothesized that promoting a large rumen by feeding more forage in early life may determine the type of microbiota harbored in the rumen and consequently the digestion efficiency of the animal.

## Factors that Influence the Microbiota Establishing in the Rumen and Long Term Effects

### Sequential Microbial Colonization of the Rumen

The gastrointestinal tract of most animals is supposed to be sterile and germ free right after birth; then, microbes from other adult animals and the surrounding environment subsequently colonize the rumen until a very complex and diverse microbial population develops (Ziolecki and Briggs, 1961). Several studies have shown that in young ruminants and during rumen development, ingested microbes colonize and establish in a defined and progressive sequence (Stewart et al., 1988). Ample evidence (Fonty et al., 1987; Morvan et al., 1994) now exists that a significant proportion of the strict anaerobes that become predominant in the mature rumen are already present in the rumen 1 or 2 days after birth. The use of molecular techniques has shown the complex microbial community that soon establishes in the non-mature rumen. All major types of rumen **bacteria**, including proteolytic and cellulolytic species, as well as some niche specialists, are present in the rumen microbial community of 14 days old calves (Li et al., 2012), whilst Jami et al. (2013) stated that “some rumen bacteria essential for mature rumen function could be detected as early as 1 day after birth”. Rey et al. (2013) monitored the establishment of ruminal bacterial community in dairy calves from birth to weaning. They showed that the establishment is rapid after birth and sequential: *Proteobacteria* is gradually replaced by *Bacteroidetes* as the main Phyla. Between days 3 and 12, the bacterial community was composed of many bacteria present in the developed rumen, showing that the bacteria responsible for the degradation of feeds are present before the ingestion of solid substrate begins. Between days 9 and 15, diet influence seemed strongest and was associated with a change in the bacterial community structure. From 15 days on, the community no longer exhibited clear time related changes at phyla level although variations on the relative abundance of some genera did occur (**Table 2**).

**Table 2:** Age classification of bacterial groups colonizing the rumen from birth to weaning. Values expressed as range of mean percentages^a^.

	Age (days)				
	3	7	14	28	42
**Phyla**					
Proteobacteria	46.6,70.4	16.9,18.7	6.45,16.9	1.8,27.6	12,27.6
Bacteroidetes	13.9,42.6	56.3,56.9	46,61.3	49.9,56.3	56.3,74
Firmicutes	5.05,13.9	13.9,17.5	13.9,34	13.9,42.1	10,13.9
Actinobacteria	0.05,4.9	0.55,4.9	0.95,4.9	0.25,4.9	4.9
Fusobacteria	4.7,5.55	4.7,5.30	0.2,0.55	0.2,0.3	0.2,0.4
Spirochaetes	0,0.4	0.1,0.4	0.4,2.60	0.4,0.85	0.4
Fibrobacteres	0,0.3	0,0.3	0.2,0.3	0.3,1.45	0.3,1.6
Tenericutes	0	0.80	0.20	0.90	0.95
Elusimicrobia	0	0	0.20	1.45	2.1
Lentisphaerae	0	0	0.15	0.20	0.31

*^a^Data collected from Li et al. (2012), Jami et al. (2013) and Rey et al. (2013)*.

Becker and Hsiung (1929) first demonstrated that the rumen **ciliate protozoa** are passed from animal to animal by direct transfer of saliva containing the active organisms as there is no resistant phase or cysts in their life cycle (Strelkov et al., 1933). Ciliate protozoa can normally be seen in the rumen of young ruminants within 2 weeks of birth with small entodinia established before large endomorphs and holotrich protozoa (Eadie, 1962). However, if animals are isolated from other ruminants shortly after birth no protozoa establish (Bryant and Small, 1960; Eadie, 1962), a property that has been widely used and continues to be used to study the role of protozoa in the rumen (Belanche et al., 2014).

**Methanogenic archaea** have been found in the undeveloped rumen of lambs well before the arrival of solid substrate to the rumen (2–4 days) and reach concentrations equivalent to those in adult animals around 10–14 days after birth (Fonty et al., 1987; Morvan et al., 1994). The development of molecular techniques allowed the detection of methanogenic archaea at earlier stages as probably they could not be detected by classical microbial counting (Gagen et al., 2012). Guzman et al. (2015) has recently reported that at day 0 of life *M. mobile*, *M. votae*, and *Methanobrevibacter* sp. were detected in the rumen of neonatal dairy calves.

As reviewed by Stewart et al. (1988), anaerobic **fungi** established in the rumen of flock-reared lambs by 8–10 days after birth (Fonty et al., 1987). They were found in all lambs by 3 weeks of age and interestingly then were no longer detectable in 9 of the 11 lambs studied when a diet based on concentrate was provided. The fungal population was mainly composed of *Neocallimastix frontalis*; *Sphaeromonas communis* was found only sporadically. The early appearance of these fungi is another characteristic of the rumen. These microorganisms which had only previously been found in mature ruminants or when forage-rich diets are fed (Orpin and Joblin, 1988) are apparently able to develop in the rumen before solid substrate enters the rumen.

In addition to the colonization pattern of the different microbial groups in the rumen, special attention should be paid to the microbial community associated to the rumen wall. Stewart et al. (1988) stated that the **epimural bacterial community** is established shortly after birth and soon reaches concentration equivalent to those in the adult while the diversity of this community seem to change with age (Mueller et al., 1984a; Rieu et al., 1990). Mueller et al. (1984a) described 24 morphological types of bacteria associated to the rumen wall in 1- to 10-week old lambs by using scanning electron microscopy, although only seven types, found in both the lamb and the adult, could be considered indigenous members of the epimural community. This community follows a characteristic succession, with significant changes occurring in the generic composition through the first 10 weeks of life. According to Mueller et al. (1984b), the epimural community does not appear to be markedly different taxonomically from the bacterial community of rumen contents, since most isolated strains could be placed into common rumen genera. However, recent studies conducted using molecular tools disagree with that statement. Sadet et al. (2007) using PCR-DGGE found that the epithelial community differed from that of rumen contents. As expected, the nature of the feed influenced the bacterial communities from the solid and liquid rumen phases but no diet effect was observed in the rumen epithelial profiles, suggesting a strong host effect on this bacterial population. More recently, Malmuthuge et al. (2014) reported large differences between digesta and epimural bacterial communities in the rumen of pre-weaned calves, highlighting greater abundances of *Prevotella* and lower abundances of *Bacteroidetes* in digesta compared with epimural bacterial communities. Moreover, the apparent association between the development of the mucosal bacteria community with the expression of some key immune related genes in mucosal tissue (Malmuthuge et al., 2012), suggests that future work on rumen colonization should include the study of the epimural community.

### Factors that Influence Early Life Colonization

Given that the different trophic niches in the rumen ecosystem are first occupied during early life and that a key turning point in microbial colonization is the introduction of solid feed in the diet (Rey et al., 2013), an important issue to address is whether management of the newborn alters the colonization pattern. As described in Section “Sequential Microbial Colonization of the Rumen,” there is now ample evidence of the early colonization of the rumen by anaerobic microorganisms, however, very few studies have actually compared the colonization pattern of the undeveloped rumen in the context of the *factors* that facilitate (or prevent) the colonization of some microbial groups (i.e., maternal influence, offspring reared in isolation, liquid/solid feed, use of additives, etc).

Protozoa are not essential for the normal rumen functioning (Williams and Coleman, 1992); however, the presence/absence of protozoa has been associated with the structure of different bacterial and methanogens communities and different rumen fermentation pattern (Yáñez-Ruiz et al., 2007; Belanche et al., 2014). Adult ruminants harbor distinctive protozoal populations with key species such as *Polyplastron* and *Epidinium* indicative of types A and B populations, respectively (Williams and Coleman, 1992). The introduction of *Polyplastron* into the rumen of animals harboring a type B protozoal population leads to the elimination of type B protozoa, however, within most flocks sheep exist with approximately the same number of animals harboring a type A and type B populations, clearly some unknown host factor influences the colonization of the rumen of individual sheep by protozoa (Williams and Coleman, 1992). Skillman et al. (2004) used twin lambs to identify methanogens colonizing the rumen of young lambs. The similarities between the rumen methanogen populations found in twins suggested that the dam was the main source of methanogen inoculation. The **maternal** influence has been further supported in recent studies in terms of microbial development in pre-ruminants subjected to anti-methanogenic treatments (bromochloromethane, BCM). Abecia et al. (2013, 2014a) reported that the archaeal community establishing in the rumen of kids depended on whether the doe was treated or not with BCM. This suggests that any intervention applied in the early life of young animals raised by the dams should consider applying the same treatment to the doe.

Both Abecia et al. (2014b) and Belanche et al. (2015) showed a different colonization pattern for protozoa in artificially reared animals as compared to those raised by the dams. Abecia et al. (2014b) showed that natural milk feeding via the dam vs. artificial feeding with milk replacer resulted in consistently lower pH in the developing rumen of goat kids that stayed with the mothers. They hypothesized that naturally raised kids would have consumed more concentrate at an earlier stage as a result of social feeding learning. An environment with a different pH during the development would be more beneficial for some microbial groups (Palmonari et al., 2010) and may set a different microbial population in the adult animal.

Anderson et al. (1987) showed that introducing solid feed for early weaning (3 weeks) in calves promoted greater microbial abundance in the rumen as compared to calves weaned conventionally (6 weeks), but no assessment of the composition of the microbiota was performed. Early studies (Eadie et al., 1959; Ziolecki and Briggs, 1961) reported that giving forage or forage and concentrate around weaning determined the concentration of some anaerobic bacteria (lactobacilli and lactate-utilizing cocci), although no information on the persistency of such effect was provided. Yáñez-Ruiz et al. (2010) reported that feeding forage vs. concentrate around weaning modified the bacterial population colonizing the rumen of lambs and that the effect persisted over 4 months, suggesting the possibility of further exploring the feasibility of manipulating the microbial populations present in the adult animal using diets or dietary additives fed early in life.

In addition to the introduction of solid diet around weaning, nutritional interventions in early life may include (i) the direct inoculation of specific microorganisms or (ii) the use of compounds (i.e., additives) that prevent or facilitate the colonization of some microbial groups. Feeding live microorganisms to ruminants is not a novel concept and extensive work has been published on the use of ‘direct-fed microbials’ (DFM; Martin and Nisbet, 1992; Jeyanathan et al., 2014). Theodorou et al. (1990) reported that the addition of the anaerobic rumen fungus *Neocallimastix* sp increased intake and liveweight gain in calves at weaning, whilst Ziolecka et al. (1984a,b) reported that a stabilized rumen extract enhanced live weight gain and stimulated rumen development in calves during weaning and Zhong et al. (2014) demonstrated that inoculation of fresh rumen fluid into the rumen of lambs for 7 days improved average daily gain and digestibility in early weaned lambs. Nakanishi et al. (1993) found that adding lactic acid bacteria to starter diets of Holstein calves stimulated rumination and ruminal development, however, no performance benefits were observed and possible microbial changes during rumen development were not determined. Lesmeister et al. (2004) evaluated the effect of supplementing yeast (*Saccharomyces cerevisiae*) culture on rumen development and growth performance in neonatal dairy calves. Although yeast cultures are widely used in ruminant nutrition, the concept of applying them in the diet of pre-ruminants deserves further assessment. They conclude that the addition of yeast in dairy calf starter at 2% enhanced dry matter intake and growth and slightly improved rumen development. Unfortunately they did not study either the effect on the rumen microbiota or the long-term effects in the animals. Other microbes targeting the rumen (i.e., *Megasphaera elsdenii*, propionibacteria) have been used as rumen probiotics but to our knowledge only in adult animals (Klieve et al., 2003).

A different experimental approach is to provide specific microbes in gnotobotically reared neonates. Gnotobitic lambs harboring either a simple or complex microbiota are an important method for investigating the role of specific microbes in the rumen. This approach has been used mainly to gain insight in the manipulation of microbes directly involved in H_2_ transfer within the rumen. Hydrogenotrophic acetogens colonize first the rumen and then they are gradually replaced by methanogenic archaea as the rumen develops (Gagen et al., 2012). The early establishment of acetogenic and sulfate-reducing bacteria underlines the competition that exists between H_2_-utilizing species. Naturally reductive acetogenesis is not a significant hydrogen sink in the rumen. However, in the absence of methanogenesis, acetogens contribute to H_2_ capture and can sustain functional rumen. Fonty et al. (2007) demonstrated using gnotobiotic lambs, that in animals lacking ruminal methanogens, the introduction of acetogens made reductive acetogenesis the major hydrogenotrophic process and that the effects of such intervention applied after birth persisted 12 months later. They suggested that if reliable methods for eliminating methanogens from early life and maintained the inoculation with acetogens could be a feasible option to decrease methane emissions from adult animals. More recently, Gagen et al. (2012) used lambs that were born naturally, left with their dams for 17 h and then placed into a sterile isolator and reared aseptically. They were inoculated with cellulolytic bacteria and later with *Methanobrevibacter* sp.7 to investigate the effect of methanogen establishment on the rumen acetogen population since they lacked cultivable methanogens. Methanogens were present in lambs isolated 17 h after birth, though were undetectable using traditional cultivation techniques. Methanogen numbers were low in these lambs (<10^4^ rrs copies per microgram of DNA) however, mcrA diversity was not dissimilar to that found in 2-year-old conventional sheep. This suggests that early colonizing methanogens may persist in the rumen and supports the potential of early life microbial programming.

With regards to the suppression of methanogens in early life, the use of compounds that inhibit the establishment of certain microbial groups or favor the development of others is now starting to attract attention. Abecia et al. (2013, 2014a) showed that application of BCM to young goat kids modified archaeal colonization of the rumen, which was linked to a reduction in methane emission of around 50%, with the effects persisting for 3 months after weaning and cessation of treatment in kids raised by does that received the same treatment as the kids.

### Timing for Interventions in Early Life and Persistency of the Effects

Given that particular factors favor the establishment of certain microorganisms, we still need to know what the most sensitive window of time for interventions is in early life. Recent work (Rey et al., 2013; Abecia et al., 2014b; Guzman et al., 2015) showed that initial colonization occurs straight after birth and that it takes 3–4 weeks for the bacterial community structure to reach a certain degree of stabilization from birth, suggesting that this period is critical. However, this assumes that once the community is more or less constant, there is no room for ‘programming’ and this has not yet been fully confirmed. It is clear that nutrient supply and hormonal signals at specific times during development (both pre- and early post-natal) exert permanent changes in the metabolism of humans (Fall, 2011), as well as changes in performance, body composition, and metabolic function of the offspring of livestock (Wu et al., 2006). These changes occur through processes generically referred to as fetal programming and metabolic imprinting. The information available in ruminants suggests that microbial colonization occurs earlier than functional achievement (i.e., a functioning rumen), with anatomic development occurring last (Jiao et al., 2015). However, the actual window of time in which such changes can be exerted in relation to microbial colonization and more importantly the persistency of the imprint needs to be further clarified. Therefore, there is urgent need to further address this question with more fundamental research. Rey et al. (2013) showed substantial colonization by the main bacterial groups in the first days of life. Likewise, Guzman et al. (2015) reported the presence of methanogenic archaea and fibrolytic rumen bacteria at day 0 in neonatal dairy calves, which suggests that the window for intervention starts straight after birth. Along these lines, there are some reported cases of human twins that harbor different gut microbiota (Clemente et al., 2012), which also offers promise for the potential of early life programming interventions. Studies in humans showed that early gut colonizers, such as those acquired from parents, can exert physiological, metabolic, and immunological effects for most of our lives (Faith et al., 2013).

## Host Immune Response to Microbiota

The gastrointestinal tract has a diverse array of non-specific and specific protective mechanisms to allow it to coexist with resident microbiota (Hooper et al., 2012). The functions of nutrients absorption, symbiotic microbial tolerance and pathogenic microbial barrier, create a conflict in function requiring a complex system of physical, biochemical, and cellular mechanisms for protection of the intestinal mucosa against invading pathogens (Kuhn and Stappenbeck, 2012).

**FIGURE 1:**
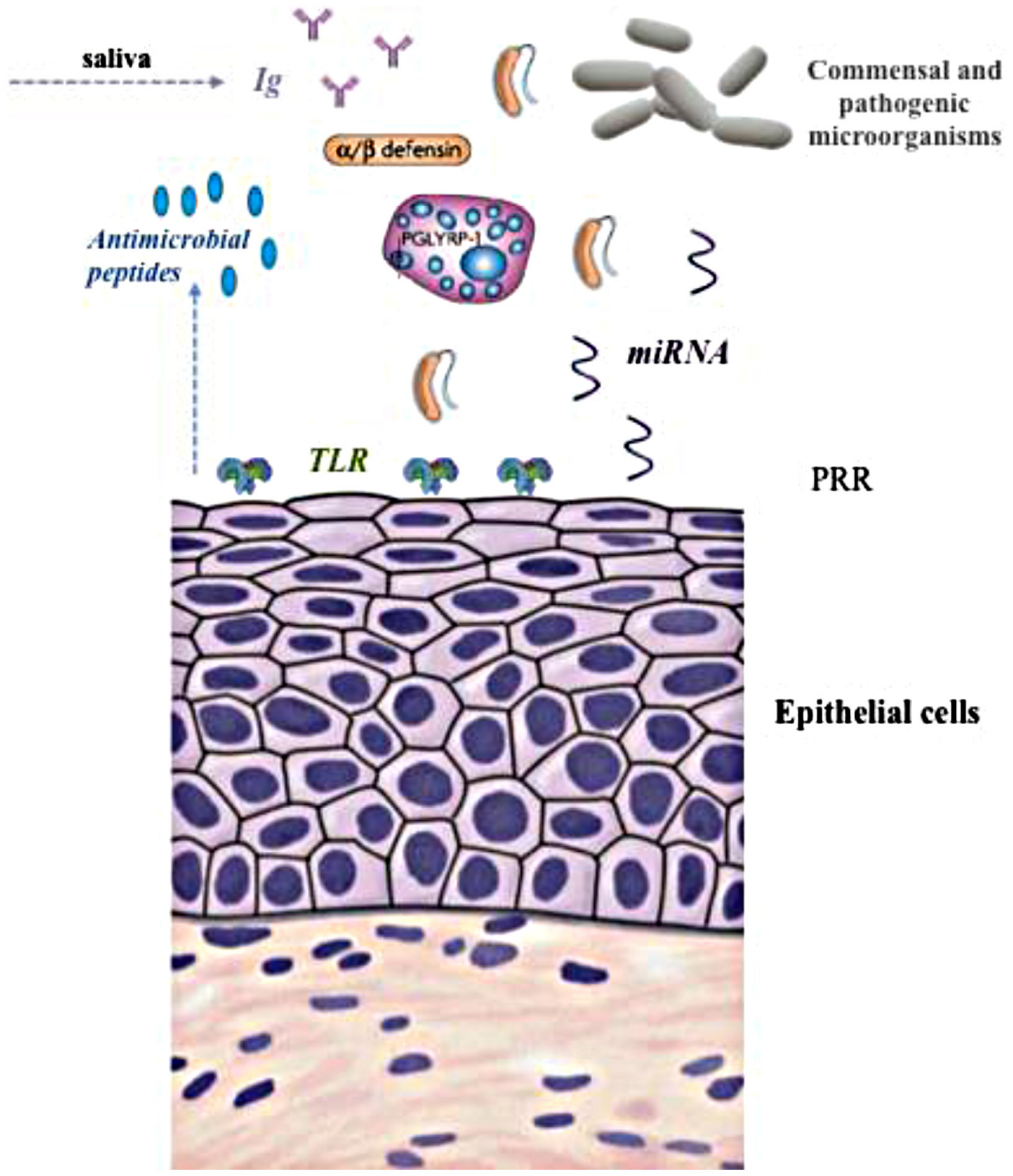
**Illustration of different elements of the immune-regulation of the rumen microbiome**. TLR, Toll-like receptor; PRR, pattern recognition receptors.

The training or education process that the immune system needs to go through to learn how to deal with microbial loads has been widely highlighted (Wu and Wu, 2012) and this is of particular importance during early life stages (Collado et al., 2012); however, the mechanisms involved in the ‘tolerance’ to the first colonizers of the rumen are largely unknown. The physiological elements involved in the case of the rumen may differ from other parts of the gastrointestinal tract due to the nature of the fermentation and the constant exposure of the rumen wall to microbial biomass. The forestomachs of the ruminant species are expanded esophageal portions lined by stratified squamous epithelium. As stated by Trevisi et al. (2014), scant information is available about the organization of the epithelial immune system in forestomachs as opposed to the impressive amount of data about the intestinal tract of both ruminant and non-ruminant species (Dommett et al., 2005). In general, the immune response in the mucosal areas of the gut is orchestrated by mucosal-associated lymphoid tissue (MALT) and gut-associated lymphoid tissue (GALT) in the gut. However, in the rumen no organized lymphoid tissue exists in the epithelium (Sharpe et al., 1977). The rumen epithelium includes up to a 15 cell layer, which can limit the permeability of large molecules. Therefore, the microbial equilibrium in the rumen is achieved by a combination of different mechanisms, illustrated in **Figure 1**: (i) constant supply of Immunoglobulins (IgA and IgG) via saliva (Williams et al., 2009), (ii) the activity of Toll-like receptors (TLRs, Malmuthuge et al., 2012), (iii) a group of genetically encoded pattern recognition receptors (Seabury et al., 2010); (iv) peptidoglycan recognition proteins (PGLYRP1, Malmuthuge et al., 2012), and (v) antimicrobial peptides defensins (Malmuthuge et al., 2012; Meade et al., 2014).

Blood serum in animals contains circulating antibodies to a wide range of Gram-negative bacteria, particularly enterobacteria (Landy and Weidanz, 1964). The antibodies are considered to be natural antibodies produced in the absence of overt infection (Boyden, 1966). Sharpe et al. (1969) found antibodies against specific strains of rumen bacteria in the blood of cows, sheep, goats, and horses, but not in pigs, rabbits, and humans. They showed the high specificity of the natural agglutinating antibodies in ruminants in absorption tests, which was further confirmed by the absence of agglutinins against a human *Escherichia coli* strain although they were detected against a rumen *E. coli* strain. A close relationship seems to exist between motility of the rumen microorganisms and their ability to stimulate natural antibodies, with antibodies being detected against motile *Butyrivibrio*, *Streptococci*, and *Lactobacilli* (Sharpe et al., 1969). Further work showed that antibodies against these organisms were present in bovine colostrum at the same level as serum and were transferred to the calf serum via colostrum (Sharpe et al., 1977). While not excluding the possibility that non-viable rumen bacteria leaving the abomasum could be a source of antigenic stimulus in the small intestine, an early study (Latham et al., 1971) investigated the caecum as an alternative site of antigenic stimulus. Many of the bacteria in the caecum are similar to those in the rumen, however, whereas the epithelium of the rumen is non-glandular and keratinized that of the caecum contains lymphatic tissue and plasma cells. The role of the caecum as active immune organ needs to be further studied.

Sharpe et al. (1977) used four gnotobiotic lambs, reared on milk, a starter ration and then grass cubes to understand the relationship between rumen microbial colonization in early life and antigen production. The lambs were inoculated with strains of *Veillonela*, *Prevotella ruminicola*, *Ruminicoccus*, *Selenomonas, Megasphaera, Lactobacillus, Butyrivibrio* and, in one case, *E. coli*. *Prevotella, Selenomonas*, and *Megasphaera* gave a strong immunological response, with antibodies to the former bacteria appearing at 20–40 days after inoculation and to the *Megasphaera* at 28–74 days. Agglutinins to *Veillonella* and *Ruminococcus* were weak and appeared only at 100–136 days after inoculation. As expected, no agglutinins were detected against non-inoculated bacteria. The gnotobiotic lambs did not receive colostrum and were born with only traces of immunoglobulins, but after 74–77 days had synthesized appreciable amounts of IgM and relatively little IgG. Since the primary antigenic response of an animal is to produce IgM, the preponderance of IgM is not surprising. At 140 days, IgG levels had risen considerably to similar levels as IgM. These results highlighted the strong link between rumen microbial colonization and specific antigen production. Unfortunately, no further work has been conducted in animals reared under different conditions in early life.

As noted earlier, saliva seems to be the main vehicle of introducing immunoglobulins in to the rumen. The levels of IgA and IgG in cattle serum, saliva, and rumen fluid have been studied recently in the context of exploring the possibility of vaccinating ruminants against specific rumen microorganisms (Subharat et al., 2015). These studies have confirmed that the major class of immunoglobulin in bovine saliva is IgA and showed that this class of immunoglobulin is also the dominant type in the rumen. In contrast, in serum the major class is IgG. IgA is apparently more resistant to degradation in the rumen compared to IgG, possibly because the secretory component of IgA makes the immunoglobulin more resistant to protease activity in the rumen (Snoeck et al., 2006). Although the research conducted in developing vaccines against specific rumen microorganisms proves that an increase in the titres of Ig in saliva can be achieved, the role of the constant supply of Ig into rumen through saliva in shaping the commensal microbial community and how this innate response functions during rumen development are as yet unknown.

A change in the diet of the animal can result in a shift in the proportion of microbial groups in the rumen (Petri et al., 2013) but little is known as to how the immune system deals with such a shift in ruminants. Some research has been conducted in this area in animals subjected to rumen acidosis challenge. Chen et al. (2012) reported a significant variation of TLR4 gene expression in the rumen epithelium of the animals with different susceptibility to acidosis. The observed correlations between copy number of total bacterial 16S rRNA genes of epimural bacteria, and ruminal pH, total VFA concentration, and expression of the TLR4 gene, suggested that the innate immune response in the epithelium is associated with the activity of the epimural bacteria. Currently, a total of 10 TLRs have been described in ruminants (Seabury et al., 2010) and two main groups may be distinguished: (i) TLRs1, 2, 4–6, 10, which are expressed in the cell surface and identify bacterial surface associated molecular patterns and (ii) TLRs3, 7–9 that recognize specific nucleic acids from viruses and bacteria (Chang, 2010; Guan et al., 2010; Malmuthuge et al., 2012). In spite of the knowledge available on the innate epithelial-associated response in adult ruminants, very few studies have addressed this in young animals. Malmuthuge et al. (2012) studied the regional and age-dependent expression patterns of TLRs, peptidoglycan recognition protein 1 (PGLYRP1), and antimicrobial proteins (β-defensin) in the rumen, jejunum, ileum, cecum, and colon of 3 weeks and 6-month-old calves. The expression of most TLRs was significantly down regulated throughout the gastrointestinal tract with increasing age. The restricted expression of both β-defensin and PGLYRP1 prior to weaning in calves suggests that significant developmental changes occur in the epithelial immune system of cattle at this time. Malmuthuge et al. (2012) hypothesized that ‘newborns may depend on TLRs as a primary innate immune mechanism to monitor commensal microflora and pathogens prior to weaning, but with increasing age it appears that other innate immune effecter mechanisms such as antimicrobial peptides may become more active in providing host defenses and minimizing harmful inflammatory responses’. No studies, however, have been conducted yet on to what extent the expression level of TLR respond to different microbial colonization patterns.

Recently, Liang et al. (2014) studied the potential regulatory role of micro RNAs (miRNAs) in the development of gastrointestinal tract (including the rumen), during the early life of dairy calves. The first finding is that the copy numbers of 16S rRNA gene of *Bifidobacterium* or *Lactobacillus* species or both were positively correlated with miR-15/16, miR-29, and miR-196 expression levels (*P* < 0.05). The authors suggested miRNAs that were expressed differently could be regulators of the differentiation and proliferation of the cells of gastro-intestinal. Indeed Liang et al. (2014) identified three miRNAs as promoters of the gut-associated development at different levels: lymphoid tissues development (miR-196), dendritic cells maturation (miR-29), and of immune cells (miR-15/16). Their results provide novel evidence of gut development mechanisms that are regulated by host-microbiome interactions (Liang et al., 2014).

As stated earlier, the information of the impact of different colonization patterns on the immune system and the long-term effects is scarce. It could be hypothesized that if the colonization of a specific microbial group is prevented in early life, it is likely that the immune system will not recognize that group in later life if it colonizes the rumen later on. As a consequence, the host may mount an immune response against it. This might be a means to control specific populations. However, this is not entirely supported by the research conducted using protozoa-free raised lambs that were inoculated with protozoa later in life (Belanche et al., 2015). Nevertheless, more research is needed to understand the immune response in animals subjected to different microbial colonization patterns and how the animal responses later in life when it is challenged with the inoculation of ‘unknown’ species.

## Conclusion and Future Prospect

The development of the rumen needs to be understood at different levels: anatomical, functional and microbial, as they have different temporal sequences in the young animal and the interplay of anatomical/functional rumen development and microbial development is not yet clear.

Notwithstanding the knowledge gaps, from the work described above, we conclude that early life events may be related to the microbial community structure and/or the rumen activity in the animals post-weaning. This would create differences in adaptive capacity due to different early life experiences and leading to the idea of microbial programming. However, the most effective window of time for intervention and the long-term implications are yet to be addressed. Therefore there is a need to perform trials that run long enough to truly assess the impact on the productive life of the animal. In addition, the differences in animal responses in later life need to be adequately assessed. In some cases, there will be no differences between animals reared differently in early life, which nevertheless have different microbiome compositions, if they are tested under ‘standard’ feeding conditions. The potential different response might become evident when the animals are nutritionally challenged or re-treated with the same pro- or anti-microbial compound as in early life.

Identifying the key immune elements at molecular level involved in early life colonization (with special attention to the rumen epimural population) may help to understand the host-animal response and the extent of persistency of effects in adult life.

## Conflict of Interest Statement

The authors declare that the research was conducted in the absence of any commercial or financial relationships that could be construed as a potential conflict of interest.
